# m6A‐Mediated Stabilization of PRMT9 mRNA by IGF2BP1 Drives Proliferation and Metastasis in Lung Adenocarcinoma

**DOI:** 10.1155/ancp/6050688

**Published:** 2025-12-10

**Authors:** Jinghua Chen, Jiahao Yang, Zihao Chen, Xiangpeng Chu

**Affiliations:** ^1^ Department of Cancer Center, Guangzhou Twelfth People’s Hospital, No. 1 Tianqiang Road Tianhe District, Guangzhou, 510620, Guangdong, China; ^2^ Department of Pulmonary Surgery, Guangdong Provincial People’s Hospital, Guangzhou, China, gdghospital.org.cn

**Keywords:** IGF2BP1, lung adenocarcinoma, m6A modification, PRMT9

## Abstract

**Background:**

Protein arginine methyltransferase 9 (PRMT9) is dysregulated in various malignancies, particularly in lung adenocarcinoma (LUAD). This study aims to systematically investigate the expression patterns, biological functions, and underlying molecular mechanisms of PRMT9 in LUAD pathogenesis.

**Methods:**

PRMT9 expression was evaluated in paired clinical LUAD specimens and adjacent normal tissues, as well as in normal alveolar epithelial cells versus established lung cancer cell lines. Genetic silencing of PRMT9 was performed in A549 and H1568 cell models to assess its functional impact. Cellular migratory and invasive capacities were quantified using wound healing and Transwell invasion assays. The mechanism of PRMT9 overexpression was explored by examining m6A‐mediated mRNA modification. The role of IGF2BP1 was determined through loss‐of‐function and gain‐of‐function experiments, supplemented with studies under PRMT9‐deficient conditions. Downstream signaling was investigated using specific inhibitors targeting the RAS and MAPK pathways, with in vivo validation in xenograft models.

**Results:**

PRMT9 was significantly upregulated at both transcriptional and translational levels in LUAD tissues and cancer cell lines compared to normal controls. Genetic depletion of PRMT9 substantially impaired cell migration and invasion, and suppressed activation of the RAS/MEK/ERK signaling pathway. The aberrant expression of PRMT9 was mechanistically linked to IGF2BP1‐regulated m6A modification. IGF2BP1 was similarly overexpressed in LUAD specimens and cell models. Knockdown of IGF2BP1 reduced PRMT9 m6A modification, compromised cell viability, migration, and invasion, and attenuated RAS/MEK/ERK signaling. Conversely, IGF2BP1 overexpression enhanced malignant behaviors, effects that were reversed by concurrent PRMT9 knockdown. Mechanistically, PRMT9 overexpression activated the RAS/MAPK signaling axis, and pharmacological inhibition of this pathway mitigated PRMT9‐mediated metastatic progression. In vivo studies confirmed that PRMT9 suppression inhibited tumor growth, which was associated with decreased expression of RAS, pMek1/2, pErk1/2, and Ki67, alongside enhanced caspase‐3 expression.

**Conclusion:**

PRMT9 is overexpressed in LUAD and promotes malignant progression by activating the RAS/MAPK signaling cascade. This aberrant PRMT9 expression is governed by IGF2BP1‐mediated m6A modification. These findings suggest that therapeutic targeting of PRMT9 or the RAS/MAPK signaling axis may represent a promising strategy for LUAD treatment.

## 1. Introduction

Lung adenocarcinoma (LUAD), a major subtype of lung cancer, has seen a notable increase in incidence globally, and particularly among females and nonsmokers. LUAC currently accounts for ~40% to 50% of all lung cancer cases and typically arises from the bronchial mucosa epithelium, predominantly in peripheral regions of the lungs [1]. Early‐stage LUAD often lacks distinct clinical symptoms but then progresses to advanced stages with symptoms such as cough, hemoptysis, and dyspnea [2]. The disease progresses rapidly, with a tendency for hematogenous metastasis to occur earlier than lymphatic spread [3]. Currently, the diagnosis of LUAD relies on chest X‐rays, CT scans, and pathological examinations [4]. In recent years, the treatment methods for LUAD have diversified; however, due to its highly infiltrative and destructive growth characteristics, the overall prognosis for LUAD patients remains unfavorable.

Protein arginine methyltransferase 9 (PRMT9), a type II methyltransferase, plays an important role in regulating many biological processes by posttranslational modification of target proteins. Jiang et al. [5] reported that PRMT9 was highly expressed in tumor tissues and promoted tumor cell migration and invasion by activating PI3K/Akt/GSK‐3*β*/Snail signaling in hepatocellular carcinoma. Their subsequent research found that PRMT9 inhibits ferroptosis to accelerate hepatitis B virus‐associated hepatocellular carcinoma progression by promoting the arginine methylation of heat shock protein 8 (HSPA8) [6]. Another research team found that PRMT9 was abnormally underexpressed in osteosarcoma and could inhibit glycolysis in osteosarcoma cells by increasing the instability of hypoxia inducible factor 1 α (HIF‐1 α) [7]. In addition, a report suggested that the homozygous PRMT9 mutation (c.G40T: p.G14C) is associated with the incidence rate of LUAD [8]. Our recent findings have established that PRMT9 is functionally upregulated and promotes progression in LUAD [9]. However, the upstream regulatory mechanisms and detailed molecular pathways by which PRMT9 drives LUAD malignancy remain to be fully elucidated.

The RAS/MAPK signaling pathway is a critical regulatory pathway governing cell proliferation, survival, and differentiation. Its aberrant activation in LUAD drives tumorigenesis, disease progression, and therapeutic resistance [10]. He et al. [11] reported that downregulation of RAS/MAPK signaling pathway by drugs can inhibit malignant progression of LUAD. A study by Liu et al. [12] suggested that the KLHL17‐activated Ras/MAPK signaling pathway plays an important role in promoting lung cancer cell proliferation and metastasis. In this study, we aimed to investigate the potential correlation between PRMT9 expression and the RAS/MAPK signaling pathway.

N6‐methyladenosine (m6A) modification, as a ubiquitous and reversible chemical modification on RNA molecules, is emerging as a hotspot in the field of cancer research. Increasing evidence suggests that m6A methylation is significantly deregulated in tumorigenesis [13], metastasis [14] and cancer metabolism [12]. The m6A modification is added by “writers,” removed by “erasers,” and recognized by “readers,” that participate in mRNA export, splicing, translation, and degradation. Studies have found that dysregulation of specific m6A regulators is closely related to LUAD progression; for example, the inhibition of writer methyltransferase‐like 3 (METTL3) promotes LUAD gefitinib resistance [15]. The eraser FTO was found to be downregulated in LUAD, which destabilizes PHF1 that triggers enhanced stemness capacity [16]. The reader insulin‐like growth factor 2 mRNA binding protein 2 (IGF2BP2) was found to activate endothelial cells to promote angiogenesis and metastasis of LUAD [17]. Therefore, an in‐depth exploration of the role of m6A methylation modification in LUAD would not only assist in understanding the pathogenic mechanism of LUAD but also provide a theoretical basis for the development of novel targeted drugs.

In the present study, we found that PRMT9 was abnormally highly expressed in LUAD tissues, due to a mechanism not related to transcription regulation. Through combined screening, we also found a possible positive correlation between IGF2BP2 and PRMT9 expression. This study investigated the expression levels of PRMT9 in LUAD and its upstream and downstream regulatory mechanisms.

## 2. Materials and Methods

### 2.1. Tissue Samples and Cell Lines

A total of 30 pairs of LUAD tissue and adjacent noncancerous tissue were collected from patients undergoing surgery at Guangzhou Twelfth People’s Hospital from June 2022 to December 2023. Clinical and pathological characteristics of 30 LUAD patients are summarized in Table S1. All patients provided their signed written informed consent for study participation. All study procedures were approved by the Biomedical Research Ethics Review Committee of Guangzhou Twelfth People’s Hospital (No. 2024159). LUAD cell lines A549, HCC827, H1568, and H1975 were purchased from Wuhan Pricella Biotechnology Co., Ltd. (Wuhan, China). All cell lines were maintained in RPMI‐1640 medium containing 10% fetal bovine serum and 1% penicillin/streptomycin in a cell culture incubator (ThermoFisher Scientific, Waltham, MA, USA).

### 2.2. Cell Transfection and Treatment

To verify the role of genes in cancer cells, the coding sequences of *PRMT9* (NM_138364.4) and *IGF2BP1* (NM_006546.4) were synthesized and recombined into a pcDNA3.0 plasmid. Short chain interfering RNA specifically targeting those two genes (siPRMT9:5′‐ACGUAAUUAGAAAACUCUCUC‐3′; siIGF2BP1:5′‐UCUCUAAGCGUUUUCCUUGUA‐3′) was purchased from GenePharma (Shanghai, China). The overexpression vector or siRNA mentioned above was mixed with Lipofectamine 2000 reagent (Invitrogen, Carlsbad, CA, USA) and added to the cell culture medium for transfection into LUAD cells. In addition, the cells were also treated with inhibitors of RAS (RAS/RAS‐RAF‐IN‐1, 5.0 μM; CAS No. : 2447039‐81‐6; MCE, Monmouth Junction, NJ, USA) and the MAPK pathway (MAPK‐IN‐1, 10.0 μM; CAS No. : 2470587‐69‐8, MCE) for 24 h. To investigate the transcriptional regulation of *PRMT9*, the 2000 bp sequence upstream of the transcription start site was cloned and recombined into the pGL3‐Basic plasmid. After transfection of the recombinant plasmid into A549 and H1568 cells for 24 h, a fluorescent substrate was added and a double fluorescence assay was performed under a fluorescence microscope (Leica, Wetzlar, Germany).

### 2.3. RT‐qPCR

Tissue samples and cells (1 × 10^6^) after grouping treatment were collected, and the total RNA was extracted by using Trizol reagent (ThermoFisher Scientific). Next, 1 μg of RNA from each sample was used as a template to synthesize cDNA with the help of a reverse transcription assay kit (Beyotime, Shanghai, China). Primers targeting *PRMT9* (Forward: 5′‐ ACGACGTGAAGAATGGGCTT ‐3′; Reverse: 5′‐ CCAACCAGTTTGCAACACGA ‐3′) and *IGF2BP1* (Forward: 5′‐ TGAACACCGAGAGTGAGACG ‐3′; Reverse: 5′‐ AAGGTCTTGCAACGAGGAGA ‐3′) were synthesized by Sangon Biotech Co., Ltd. (Shanghai, China). Subsequently, quantitative PCR was performed by using a BeyoFast SYBR Green One‐Step qRT‐PCR Kit (Beyotime). The comparative threshold (CT) cycle method was used to quantify relative gene expression. The fold‐change of the experimental sample relative to the control group was estimated using the formula 2^-*ΔΔ*CT^. GAPDH served as an internal control for normalization.

### 2.4. m6A‐RIP‐qPCR

The m6A methylation of PRMT9 mRNA was detected using an m6A MeRIP kit (Ribobio, Guangzhou, China). In brief, 20 μg of RNA was added to the fragmentation buffer, mixed well, incubated at 70°C for 6 min, and then centrifuged to obtain fragmented RNA. Next, the precipitate was dissolved in 270 μL of nuclease‐free water, diluted with 30 μL of PC buffer, 1 μL of PC enhancer, and 750 μL of absolute ethanol, gently mixed, and allowed to precipitate at −20°C for 3 h. The mixture was then centrifuged at 4°C at 15,000 *g* to obtain RNA, which was subsequently washed with 75% ethanol prepared with nuclease‐free water. Next, 50 μL of preprepared IP buffer containing immunoprecipitation magnetic beads was added to 50 μL of the fragmented RNA mentioned above and supplemented with 100 μL of nuclease‐free water. The mixture was incubated on a rotating mixer at 4°C for 1 h with continuous rotation. After sequentially adding LB buffer and HS buffer and washing the magnetic beads, the final anti‐m6A‐bound RNA was obtained and used to detect PRMT9 expression.

### 2.5. Western Blotting

Total protein was extracted from tissue samples and cells (1 × 10^6^). After determining the protein concentration in each extract using a BCA kit (Beyotime Biotechnology), 20 μg of protein was loaded into each well of an SDS‐PAGE gel for electrophoretic separation. Subsequently, the separated protein bands were transferred onto a PVDF membrane (Merck Millipore, Billerica, MA, USA) by using the wet transfer method. After blocking with 5% bovine serum albumin, the membrane was incubated for 2 h with antibodies targeting IGF2BP1 (ab290736, 1:2000; Abcam, Cambridge, MA, USA), PRMT9 (ab122374, 1:1000), Ras (ab108602, 1:2000), Mek1/2 (ab278564, 1:1000), and Erk1/2 (ab278538, 1:1000), respectively. Next, the membrane was incubated with the corresponding species‐specific HRP IgG secondary antibody (Abcam), and a chemiluminescent substrate was added. Finally, the fluorescence signals of the target bands were visualized on X‐ray film (Beyotime).

### 2.6. Immunohistochemistry Assay

The tissue sample was fixed with formaldehyde, embedded in a paraffin block, and cut into 5 μm sections, which were then mounted onto glass slides. After dewaxing, hydration treatment, antigen retrieval, and blocking, each slide was incubated with a primary antibody targeting IGF2BP1 (ab290736, 1:300), PRMT9 (ab122374, 1:500), Ki67 (ab15580, 1:500) or caspase‐3 (ab184787, 1:500) at room temperature for 3 h. Next, the tissue sample on each slide was incubated with an HRP‐labeled secondary antibody for 30 min, treated with DAB solution for color development, and then counterstained with hematoxylin for 40 s. Finally, images were recorded under a microscope (Leica, Wetzlar, Germany).

### 2.7. Cell Viability Testing

Cell viability was assessed using a Cell Counting Kit‐8 Kit (Beyotime). In brief, 1 × 10^5^ cells from each group were collected and inoculated into the wells of a 96‐well plate. After adding 100 μL of CCK8 solution to each well, the plate was incubated in a 37°C incubator for 2 h. Finally, the absorbance value of each well at 450 nm was measured with a microplate reader (ThermoFisher Scientific).

### 2.8. Scratch Test

The scratch test was performed to determine cell migration ability. Cells were inoculated into a six‐well plate (~5 × 10^5^ cells/well) and cultured to ~90% confluence; after which, the cells received their specified treatments. After treatment, a 200 μL pipette tip was used to make a perpendicular scratch on each cell monolayer, and photos were recorded. The serum‐free medium was then replaced, and the cells were cultured for an additional 24 h. Finally, the scratch width was observed at the same location under a microscope.

### 2.9. Transwell Assay

To determine cell invasion, cells (5 × 10^5^) were collected and inoculated into a Transwell plate that had been precoated with a matrix adhesive. Next, 100 µL of serum‐free medium was added to the upper chamber, and 500 µL of culture medium containing 10% FBS was added to the lower chamber. The plate was then incubated for 24 h. After removing the culture medium in the lower chamber, the matrix gel and cells in the small chamber were wiped with a cotton swab or cotton soaked in PBS, and subsequently fixed with paraformaldehyde and then stained with crystal violet for 10 min. Finally, the numbers of cells in five randomly selected fields were counted under a microscope.

### 2.10. Xenograft Assay

A total of 24 female nude mice (aged 4–6 weeks; 18–22 g) were purchased from the Guangdong Medical Experimental Animal Center. shRNA targeting PRMT9 was ligated onto a lentiviral expression vector and transfected into A549 and H1568 cells. After screening for stable cell lines, ~2 × 10^6^ cells were subcutaneously injected into the forelimb axilla of nude mice. Starting at 9 days after injection, the long diameters (a) and short diameters (b) of the tumors were measured every 3 days using a vernier caliper. Tumor volume was calculated using the following formula: volume = a × b^2^/2. On Day 30, all mice were sacrificed by intraperitoneal injection of excessive sodium pentobarbital. Finally, each tumor was separated, photographed, and subjected to other tests. All procedures involving animals were approved by the Guangzhou Center for Disease Control and Prevention Experimental Animal Welfare Ethics Committee (Number 2025001).

### 2.11. Statistical Analysis

All data were analyzed using GraphPad Prism 8 software (La Jolla, CA, USA). Data with a normal distribution are presented as a mean value ± standard deviation. Student’s *t* test was used to compare differences between two groups, and one‐way analysis of variance was used for comparisons among multiple groups. A *p*‐value < 0.05 was considered to be statistically significant.

## 3. Results

### 3.1. Abnormally High Expression of PRMT9 in LUAD

To detect the expression of PRMT9 in LUAD, samples of cancer tissue and adjacent noncancerous tissue (normal lung tissue) were collected. The qPCR results showed that the expression levels of PRMT9 mRNA in cancer tissues were significantly higher than those in tissues adjacent to the cancer (Figure 1A). WB and IHC studies found that the levels of PRMT9 protein were abnormally high in the cancer tissues (Figure 1B,C). In addition, we measured the expression of PRMT9 in normal alveolar epithelial cells and lung cancer cell lines. Those results showed that the levels of PRMT9 in cancer cells, and especially in A549 and H1568 cells, were higher than those in HBE4‐E6/E7 cells (Figure 1D,E). To investigate the effect of PRMT9 expression on LUAD cancer cells, we knocked down PRMT9 expression in A549 and H1568 cells (Figure 1F,G). The scratch test and Transwell assay results showed that knockdown of PRMT9 significantly reduced cell migration and invasion abilities, respectively (Figure 1H,I). In addition, we performed WB studies to detect the activation of the RAS and Mek/Erk signaling pathways. Results showed that knockdown of PRMT9 reduced the levels of RAS, pMek1/2, and pErk1/2 proteins (Figure 1J). These results suggest that the level of PRMT9 expression may be related to LUAD progression.

Figure 1Knockdown of PRMT9 inhibited LUAD cell migration and invasion. (A) QPCR detection of PRMT9 expression levels in samples of cancer (*n* = 30) and adjacent tissues (*n* = 30). (B,C) Detection of PRMT9 protein levels by Western blotting and IHC methods. (D,E) QPCR and Western blot detection of PRMT9 expression in HBE4‐E6/E7 cells and 4 LUAD cell lines. (F,G) PRMT9 was knocked down in A548 and H1568 cells. (H) Cell migration was measured by the scratch test. ×40 magnification. (I) Cell invasion was measured by the transwell assay. ×200 magnification. (J) The levels of RAS, pMek1/2, and pErk1/2 protein were detected by Western blotting.  ^∗∗∗^ *p* < 0.001, vs. the Para‐LUAD or HBE4‐E6/E7 group. ^###^ *p* < 0.001, vs. si‐NC group. “ns” indicates *p* > 0.05, meaning the result is not statistically significant.

**Figure:**
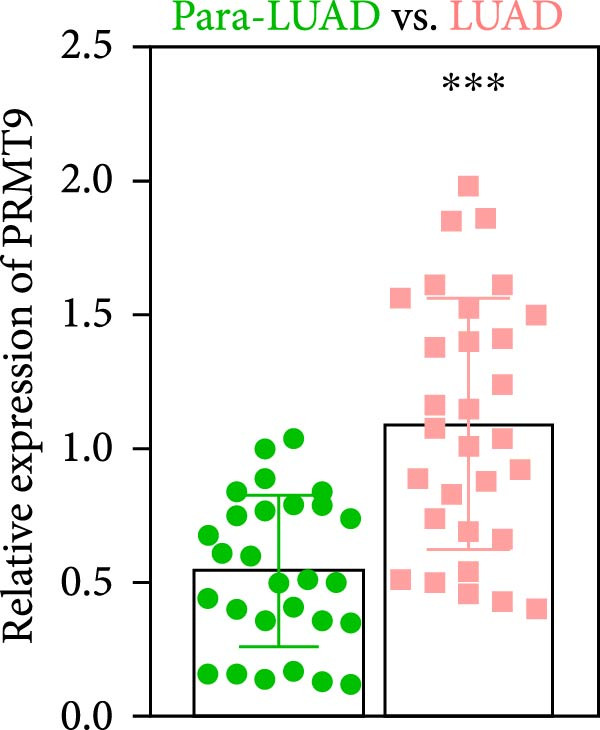
(A)

**Figure:**
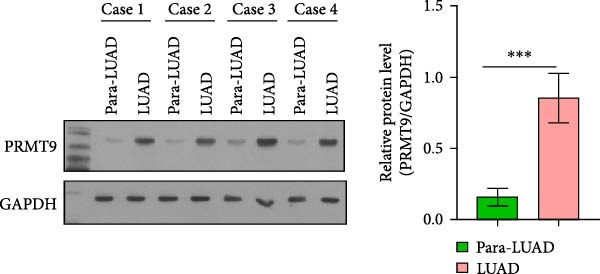
(B)

**Figure:**
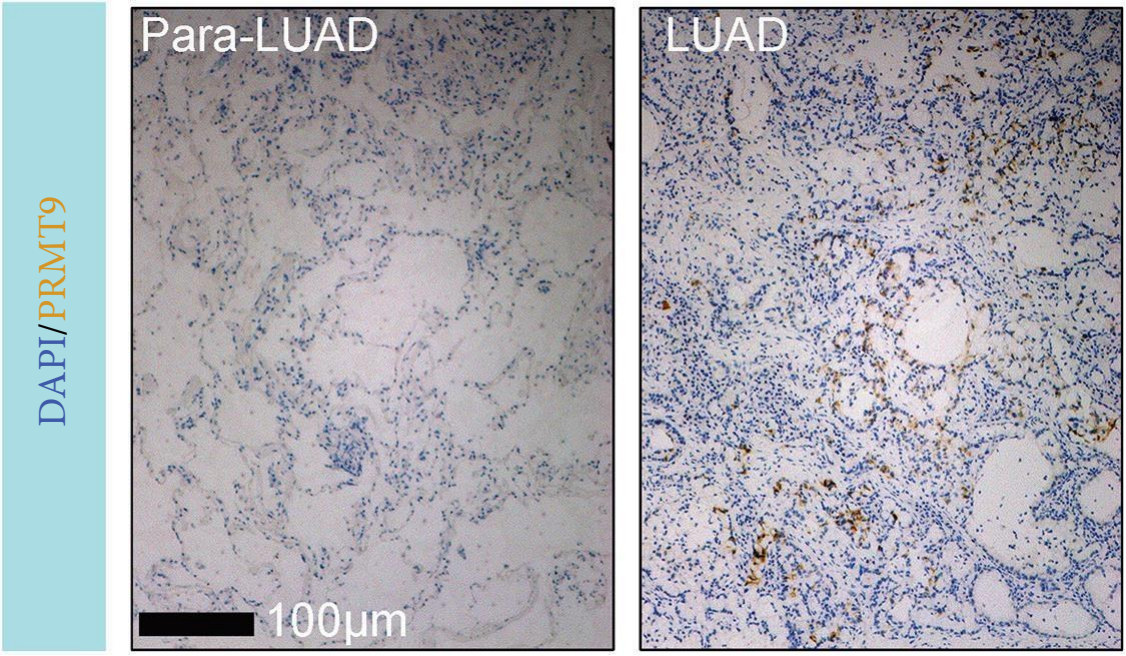
(C)

**Figure:**
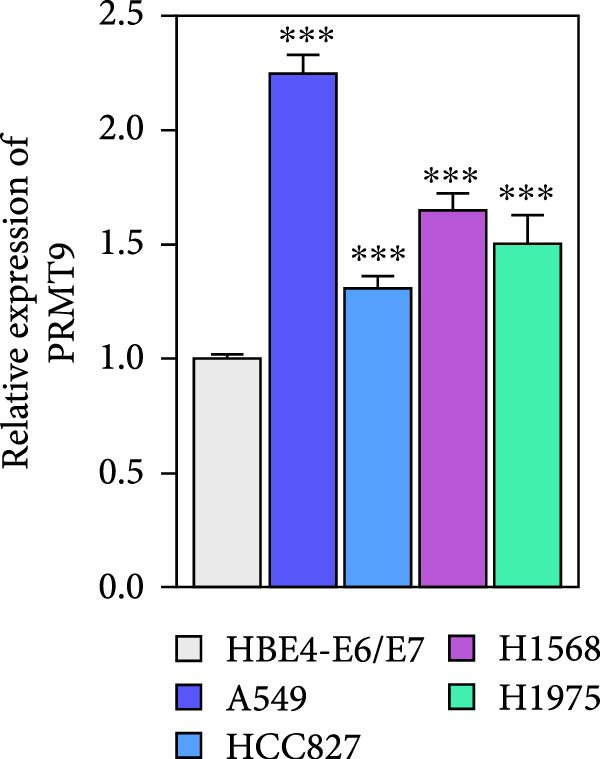
(D)

**Figure:**
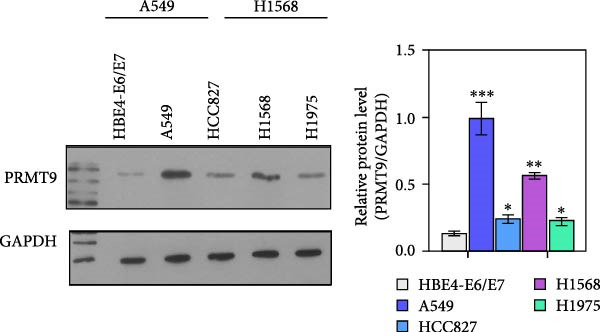
(E)

**Figure:**
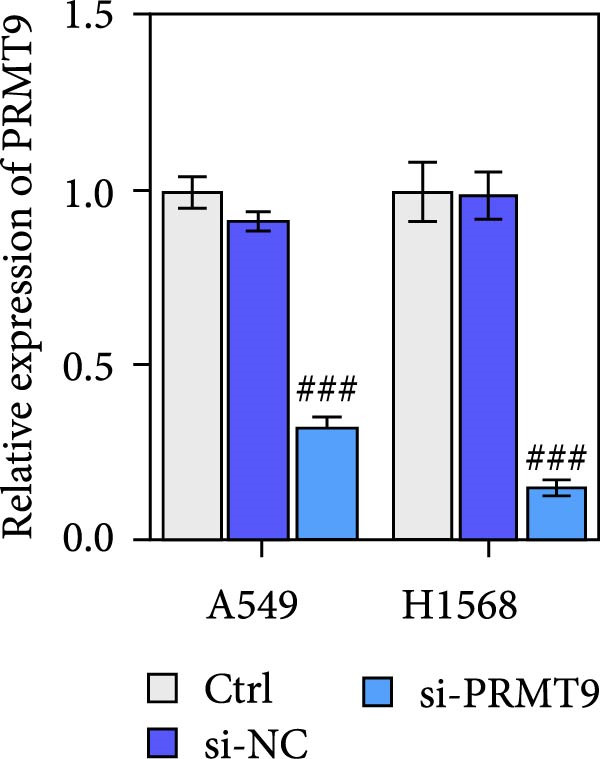
(F)

**Figure:**
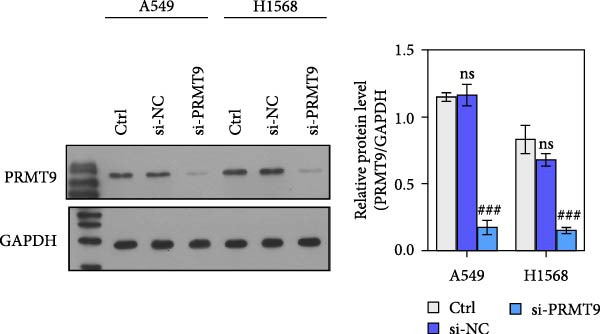
(G)

**Figure:**
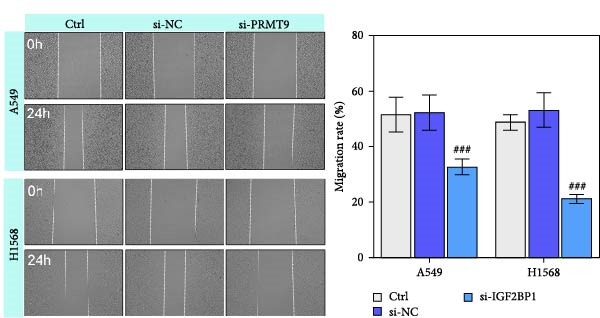
(H)

**Figure:**
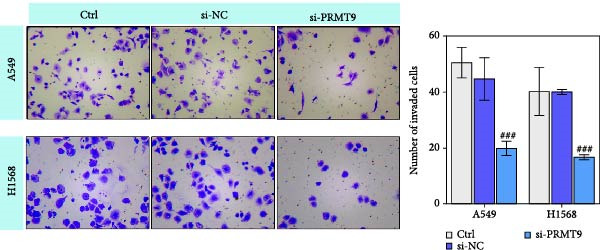
(I)

**Figure:**
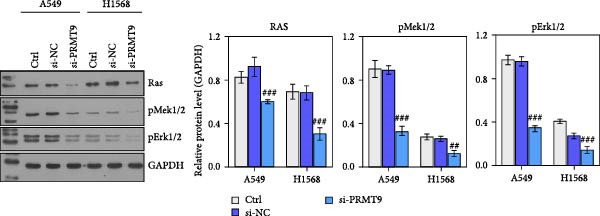
(J)

### 3.2. PRMT9 Expression was Associated With Its mRNA m6A Modification

To explore the mechanism of abnormal PRMT9 expression, we first investigated its transcriptional regulation. Results showed that the transcriptional activity of the PRMT9 promoter sequence did not differ between the HBE4‐E6/E7 cells and cancer cells (Figure 2A). However, after RIP with the m6A antibody, qPCR detection showed that the relative levels of PRMT9 mRNA in A549 and H1568 cells were significantly higher than those in HBE4‐E6/E7 cells (Figure 2B). In addition, after conducting RNA pull‐down experiments with probes targeting PRMT9 mRNA, a Western blot analysis revealed the presence of IGF2BP1 protein (Figure 2C). This suggested that PRMT9 expression levels were associated with its mRNA m6A modification, and which might be regulated by IGF2BP1. In addition, the TCGA data revealed that the expression level of IGF2BP1 was significantly elevated in LUAD tissues, and it was significantly correlated with the overall poor survival rate of patients (Figure 2D,E). It also showed a significant correlation between IGF2BP1 expression level and PRMT9 expression level (Figure 2F). Furthermore, our results showed that IGF2BP1 expression in LUAD tissues was higher than that in normal lung tissues at both the mRNA and protein levels (Figure 2G–I). The levels of IGF2BP1 expression in cancer cells were also significantly higher than those in HBE4‐E6/E7 cells (Figure 2J,K). Most importantly, after knocking down IGF2BP1 in A549 and H1568 cells, the detected levels of m6A‐modified PRMT8 were significantly reduced (Figure 2L).

Figure 2PRMT9 expression was regulated by IGF2BP1. (A) The 2000 bp upstream sequence of the transcription start site in gene *PRMT9* was cloned and recombined into the pGL3‐Basic plasmid for use in a dual luciferase reporter assay. (B) The m6A modification level of PRMT9 mRNA was detected using the RIP qPCR method. (C) Validation of IGF2BP1 binding to PRMT9 mRNA by the RNA pull down‐WB method. (D) The expression levels of IGF2BP1 in different stages of LUAD revealed by the TCGA database. (E) High expression of IGF2BP1 is significantly associated with poor overall survival in patients with LUAD. (F) Correlation analysis between IGF2BP1 expression level and PRMT9 expression level. (G) QPCR detection of IGF2BP1 expression levels in cancer (*n* = 30) and adjacent tissues (*n* = 30). (H, I) Detection of IGF2BP1 protein levels by Western blot and IHC methods. (J, K) QPCR and Western blot detection of IGF2BP1 expression in HBE4‐E6/E7 cells and 4 LUAD cell lines. (L) Knockdown of IGF2BP1 caused a decrease in PRMT9 mRNA that m6A antibodies could bind to. ns, no significant difference.  ^∗∗∗^ *p* < 0.001, vs. the Para‐LUAD or HBE4‐E6/E7 group. ^###^ *p* < 0.001, vs. the IgG group.

**Figure:**
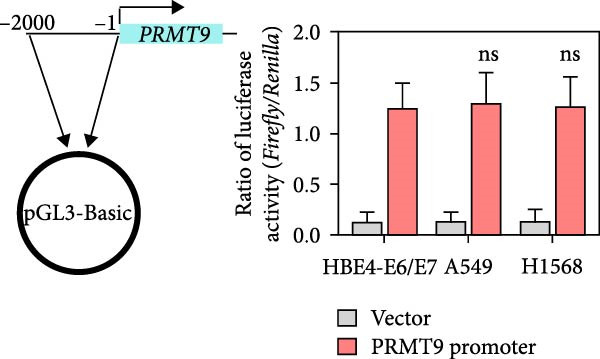
(A)

**Figure:**
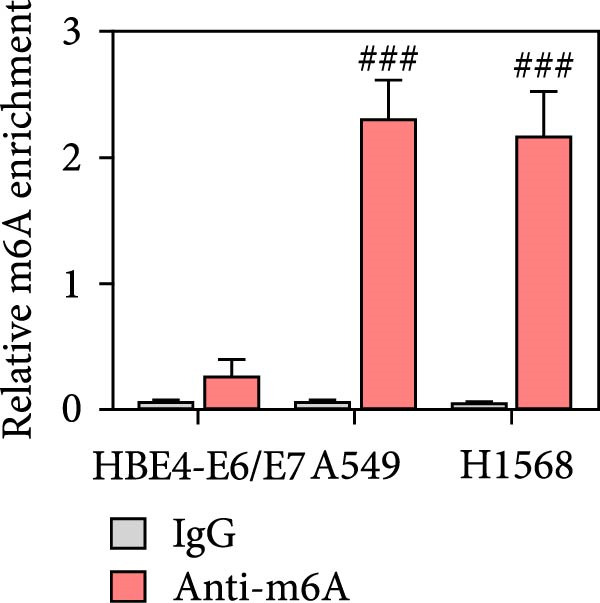
(B)

**Figure:**
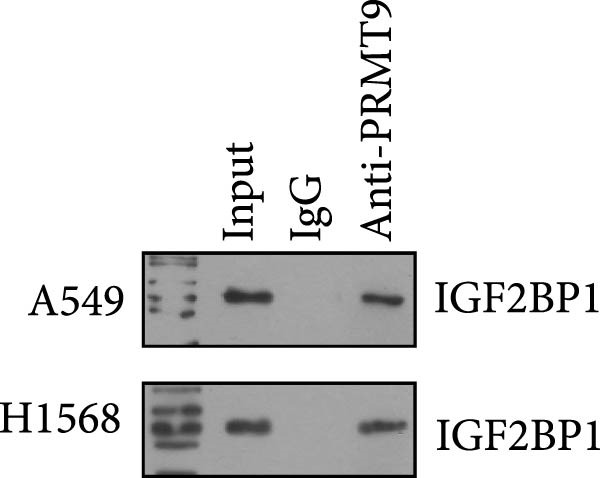
(C)

**Figure:**
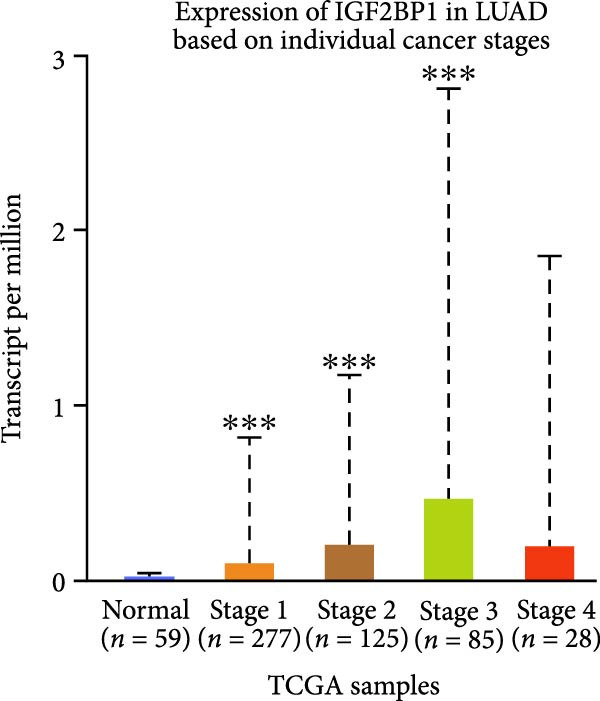
(D)

**Figure:**
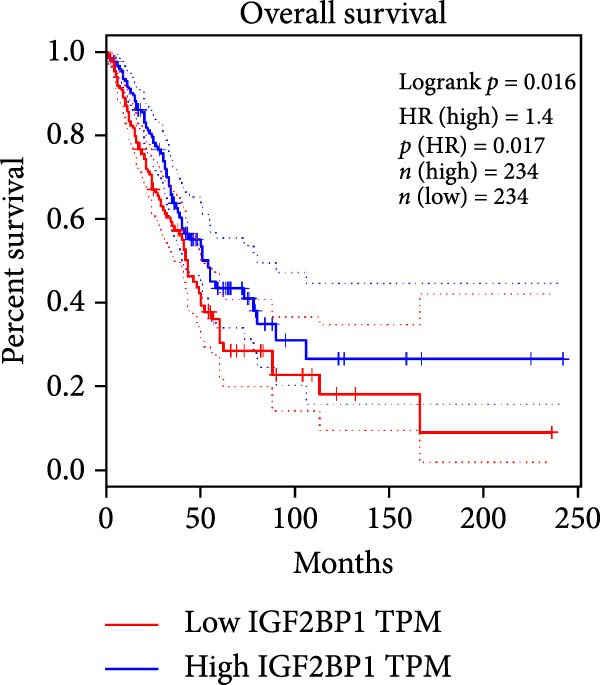
(E)

**Figure:**
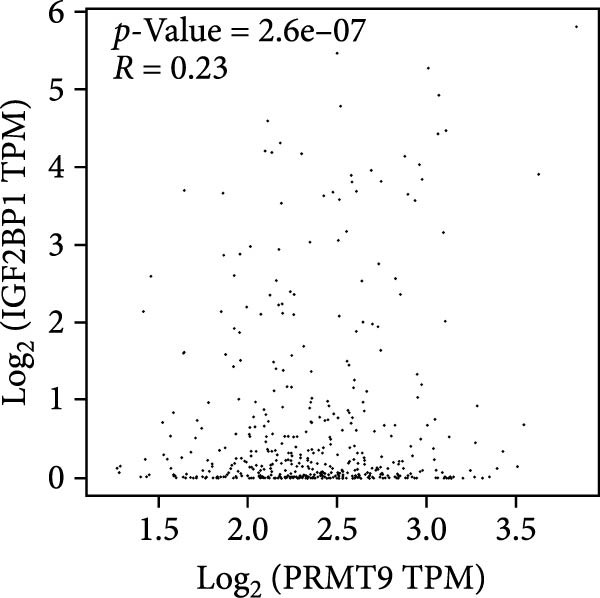
(F)

**Figure:**
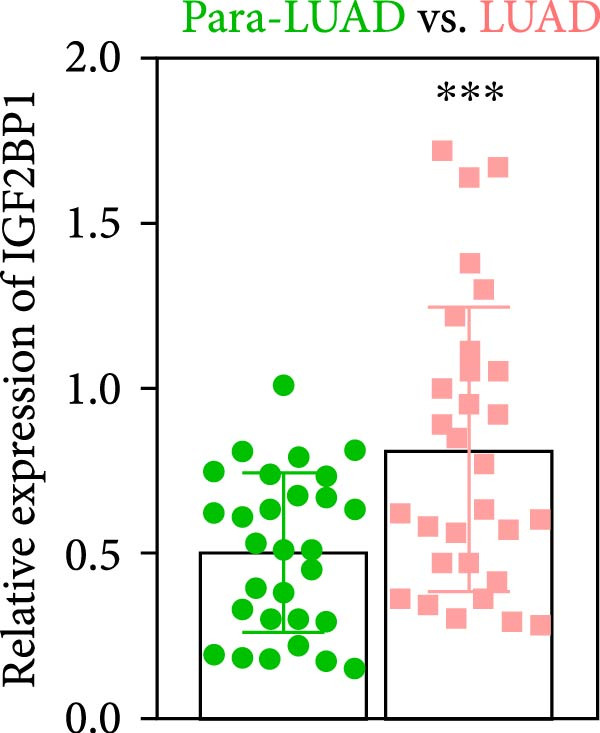
(G)

**Figure:**
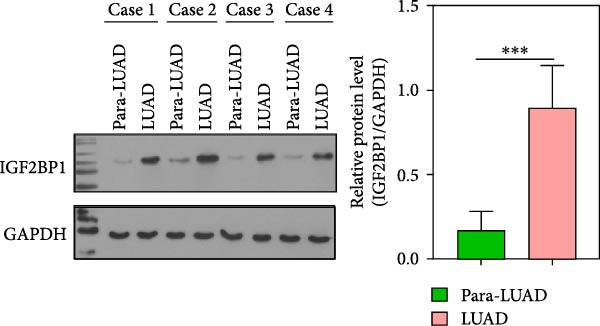
(H)

**Figure:**
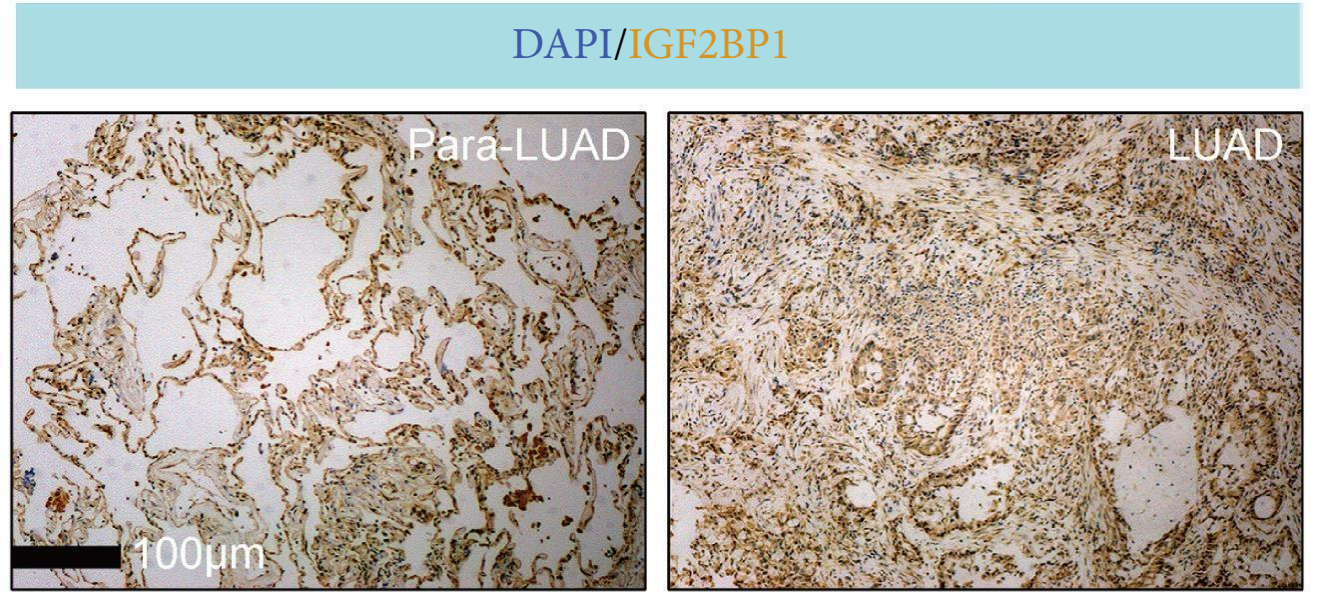
(I)

**Figure:**
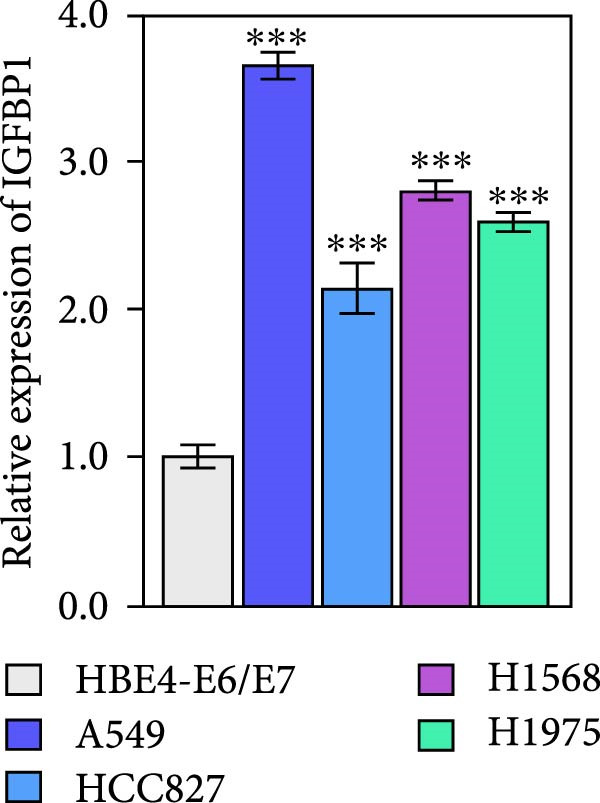
(J)

**Figure:**
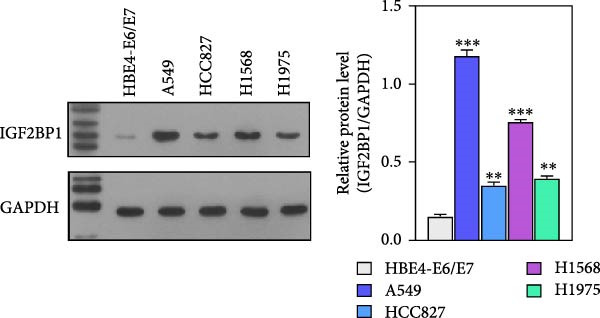
(K)

**Figure:**
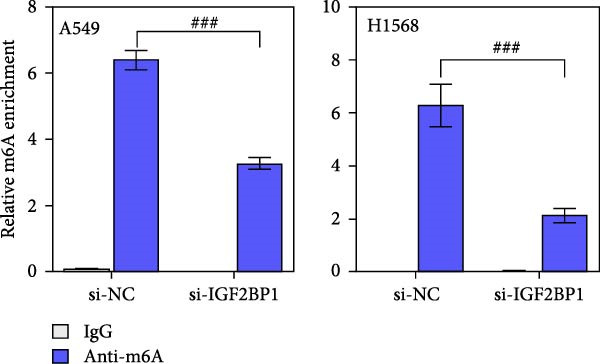
(L)

### 3.3. IGF2BP1 Promoted Cell Migration and Invasion by Regulating PRMT9

To explore the function of IGF2BP1 in LUAD, we knocked down IGF2BP1 expression in A549 and H1568 cells. QPCR and Western blot analyses showed that IGF2BP1 expression was significantly reduced in the si‐IGF2BP1 group (Figure 3A,B). CCK‐8 assays revealed that knockdown of IGF2BP1 significantly reduced cell viability after 48 h of transfection (Figure 3C). In addition, cell migration and invasion were significantly inhibited by knockdown of IGF2BP1 (Figure 3D,E), and the levels of RAS, pMek1/2, and pErk1/2 proteins were reduced (Figure 3F).

Figure 3IGF2BP1 knockdown suppresses proliferation, migration, and invasion in LUAD cells. (A, B) QPCR and western blot methods for detecting IGF2BP1 expression at 48 h after transfection. (C) Cell viability was detected by the CCK8 assay. (D) Cell migration was measured by the Scratch test. x40 magnification. (E) Cell invasion was measured by the transwell assay. ×200 magnification. (F) The levels of RAS, pMek1/2, and pErk1/2 protein were detected by Western blotting. ^###^ *p* < 0.001, vs. si‐NC group.

**Figure:**
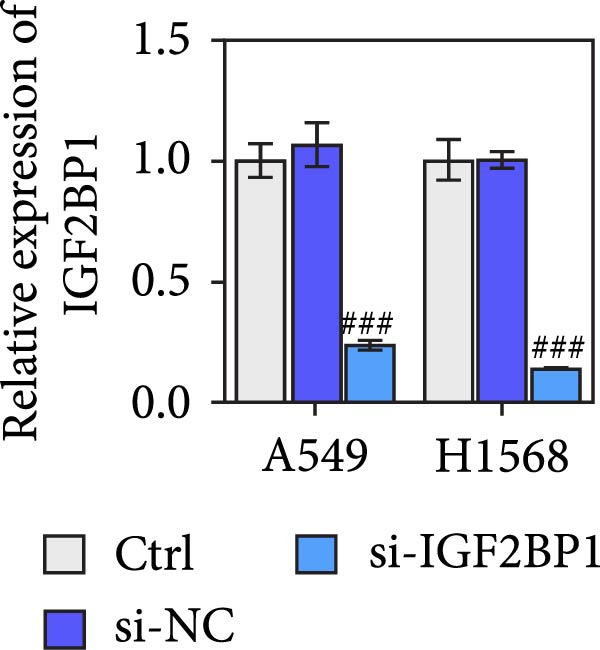
(A)

**Figure:**
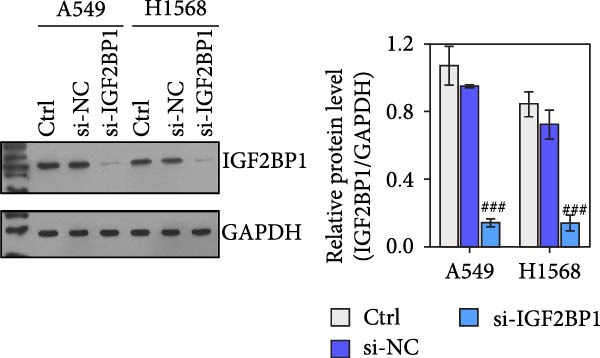
(B)

**Figure:**
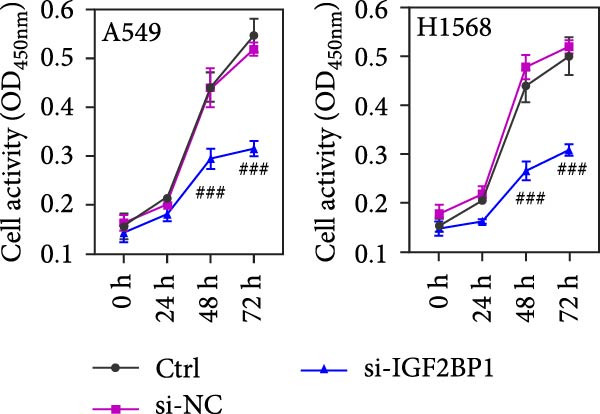
(C)

**Figure:**
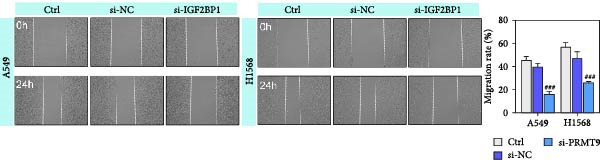
(D)

**Figure:**
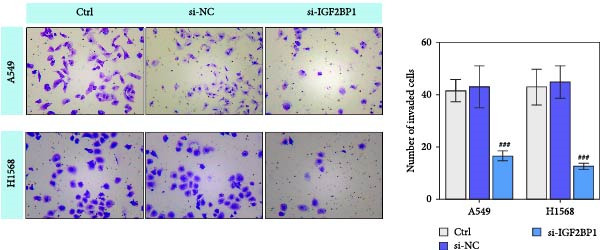
(E)

**Figure:**
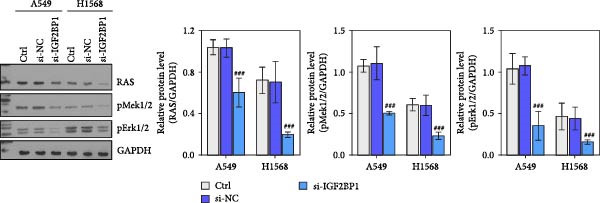
(F)

Moreover, we also overexpressed IGF2BP1 and knocked down PRMT9 expression in A549 and H1568 cells. Results showed that overexpression of IGF2BP1 significantly increased IGF2BP1 and PRMT9 expression (Figure 4A–C), while knockdown of PRMT9 had no effect on IGF2BP1 expression. In addition, overexpression of IGF2BP1 led to significant increases in cell viability, migration, and invasion (Figure 4D–F), and those effects were all reversed by knockdown of PRMT9. In the same way, the accumulations of RAS, pMek1/2, and pErk1/2 proteins induced by IGF2BP1 overexpression were also reduced by knockdown of PRMT9 (Figure 4G). These results indicated that IGF2BP1 promotes cell migration and invasion by regulating PRMT9.

Figure 4Knockdown of PRMT9 reversed the effect of IGF2BP1 overexpression on proliferation and cell migration and invasion. (A–C) QPCR and Western blot methods for detecting IGF2BP1 and PRMT9 expression at 48 h after transfection. (D) Cell viability was detected by the CCK8 assay. (E) Cell migration was measured by the scratch test. x40 magnification. (F) Cell invasion was measured by the transwell assay. ×200 magnification. (G) The levels of RAS, pMek1/2, and pErk1/2 proteins were detected by Western blotting.  ^∗∗∗^ *p* < 0.001, vs. Vector + si‐NC group; ^###^ *P* < 0.001, vs. IGF2BP1 + si‐PRMT9 group.

**Figure:**
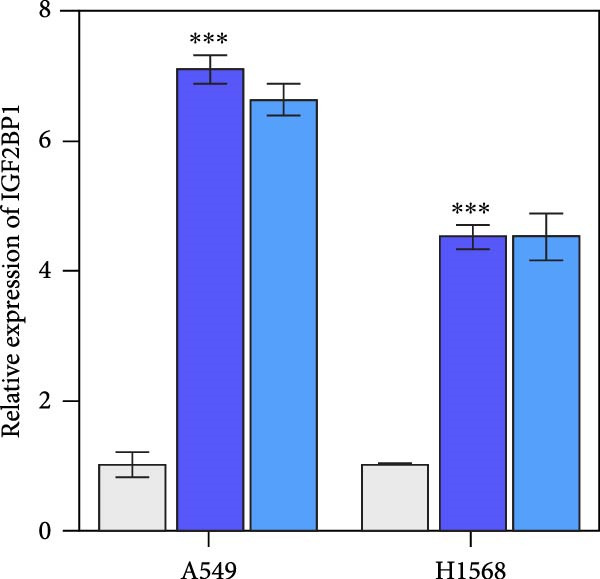
(A)

**Figure:**
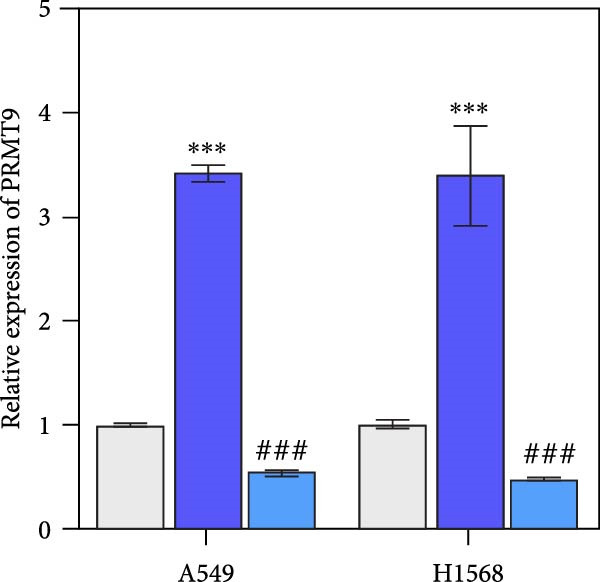
(B)

**Figure:**
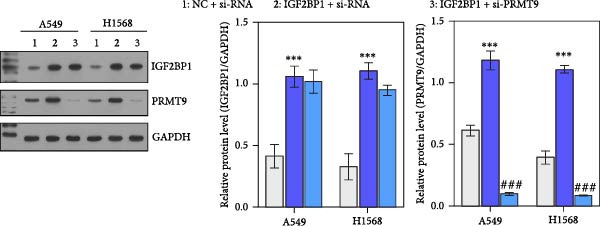
(C)

**Figure:**
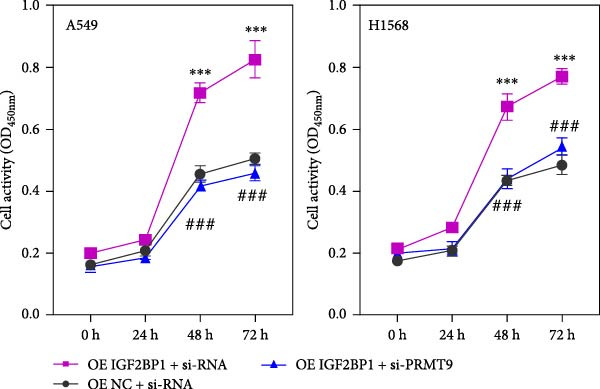
(D)

**Figure:**
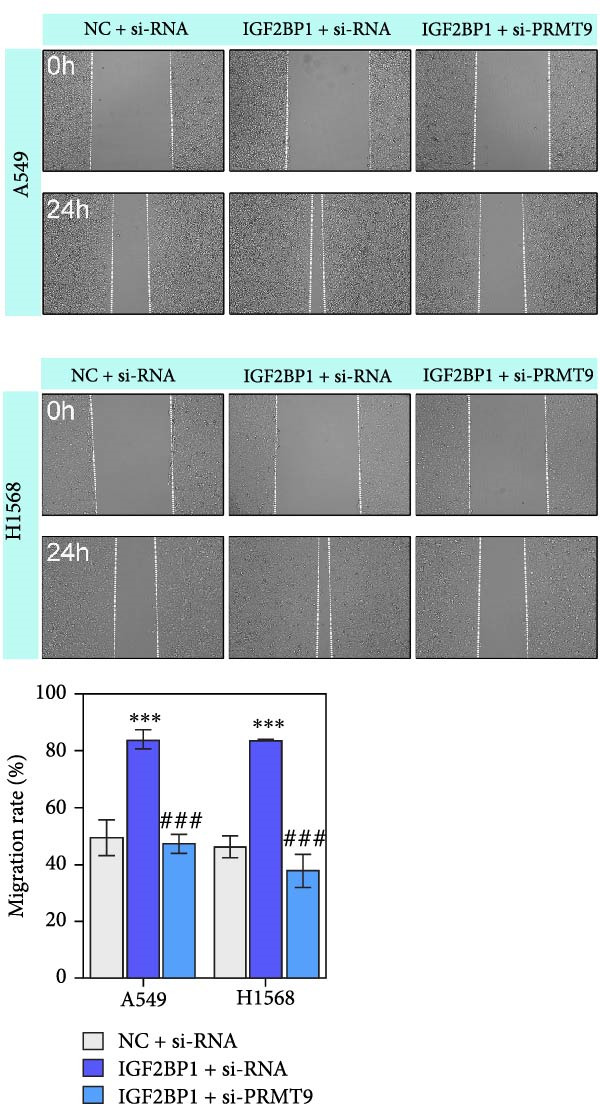
(E)

**Figure:**
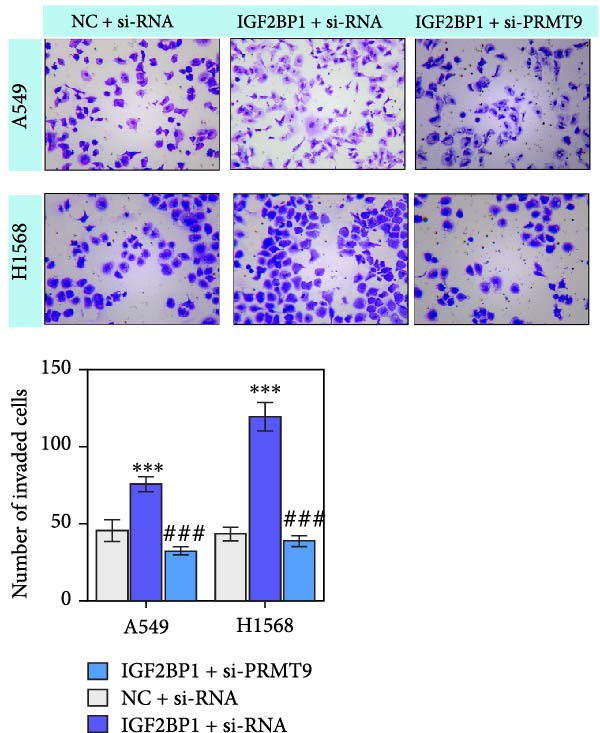
(F)

**Figure:**
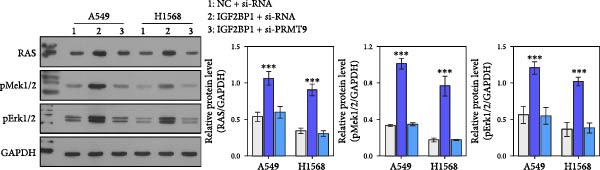
(G)

### 3.4. PRMT9 Overexpression Activated the RAS/MAPK Signaling Pathway

To explore the downstream mechanism of PRMT9, we overexpressed PRMT9 and then treated the cells with an RAS inhibitor (RAS‐IN‐1) or MAPK inhibitor (MAPK‐IN‐2). Results showed that the two inhibitors had no effect on the efficiency of PRMT9 overexpression (Figure 5A,B). However, overexpression of PRMT9 significantly increased cell viability, migration, and invasion (Figure 5C–E). More importantly, both of the inhibitors reversed the effects of PRMT9 overexpression, suggesting that blocking the RAS/MEK/ERK signaling pathway could attenuate the effect of PRMT9 on LUAD metastasis. Moreover, a Western blot analysis also confirmed the effect of PRMT9 overexpression on the RAS/MEK/ERK signaling pathway (Figure 5F). In vivo studies revealed that knockdown of PRMT9 significantly inhibited tumor growth (Figure 6A,B). In tumor tissue samples, knockdown of PRMT9 decreased the levels of RAS, pMek1/2, Ki67, and pErk1/2 proteins (Figure 6C), while caspase‐3 levels were increased by knockdown of PRMT9 (Figure 6D). Both in vivo and in vitro experiments suggested that LUAD progression promoted by PRMT9 may be related to the RAS/MAPK signaling pathway.

Figure 5Blocking the RAS/MAPK pathway reversed the effect of PRMT9 overexpression on proliferation and cell migration and invasion. (A, B) QPCR and Western blot methods for detecting PRMT9 expression at 48 h after treatment. (C) Cell viability was detected by the CCK8 assay. (D) Cell migration was measured by the scratch test. x40 magnification. (E) Cell invasion was measured by the transwell assay. ×200 magnification. (F) The levels of RAS, pMek1/2, and pErk1/2 proteins were detected by Western blotting.  ^∗∗∗^ *p* < 0.001, vs. Ctrl group; ^###^ *p* < 0.001, vs. PRMT9 group.

**Figure:**
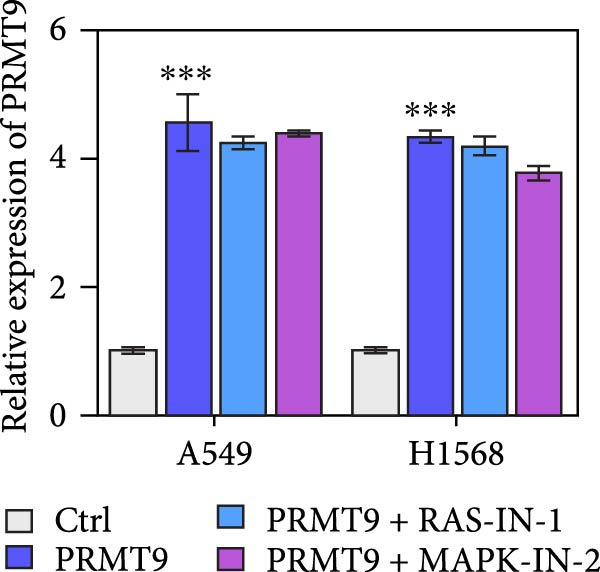
(A)

**Figure:**
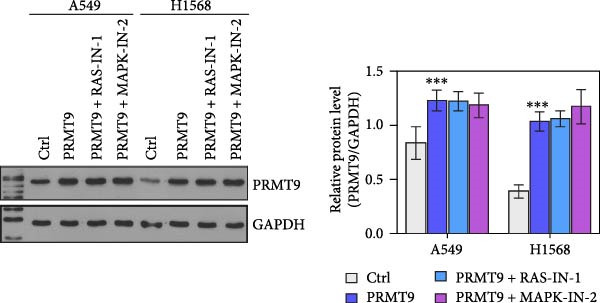
(B)

**Figure:**
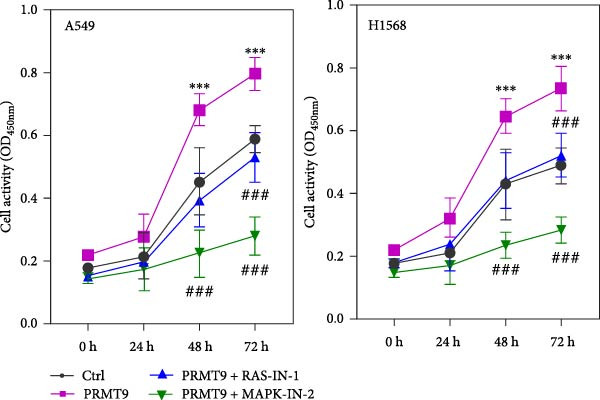
(C)

**Figure:**
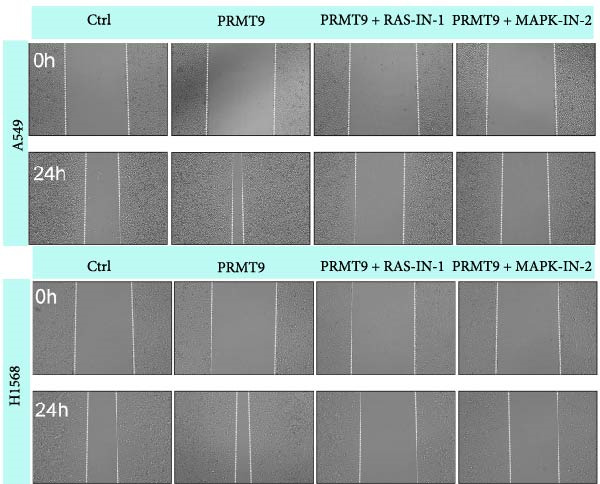
(D)

**Figure:**
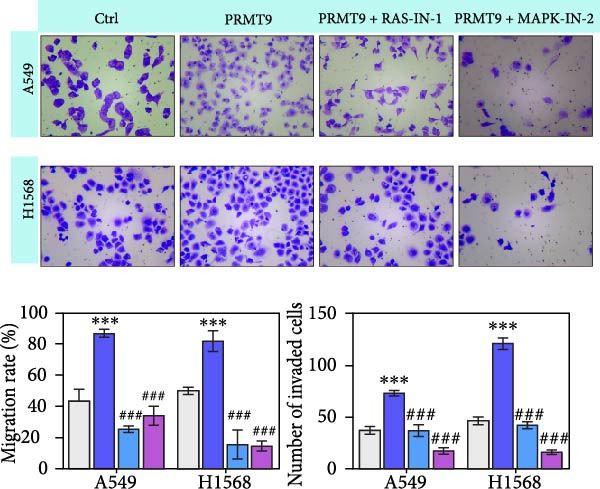
(E)

**Figure:**
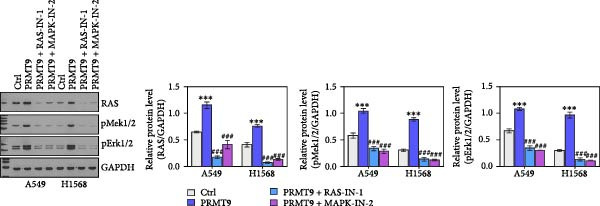
(F)

Figure 6In vivo experiments confirmed that knockdown of PRMT9 inhibited tumor growth. (A) Images of tumors in mice and tumors in vitro after excision. (B) Starting from day 9 after injection, the calculated tumor volume was measured every 3 days. (C) The levels of PRMT9, RAS, pMek1/2, and pErk1/2 proteins were detected by Western blotting. (D) The expression levels and localization of Ki67 and caspase‐3 in tumors as shown by the IHC assay.

**Figure:**
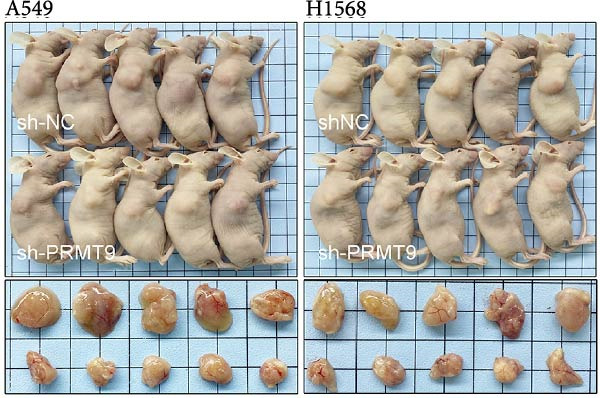
(A)

**Figure:**
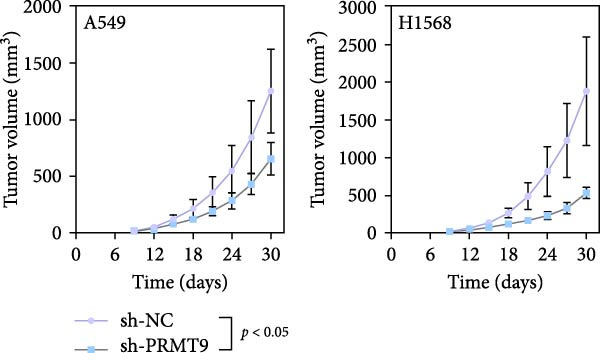
(B)

**Figure:**
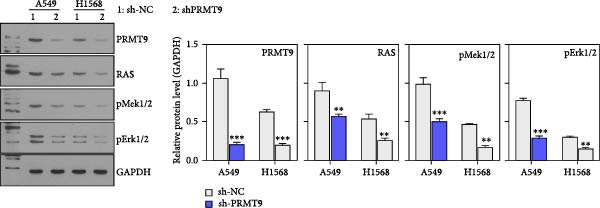
(C)

**Figure:**
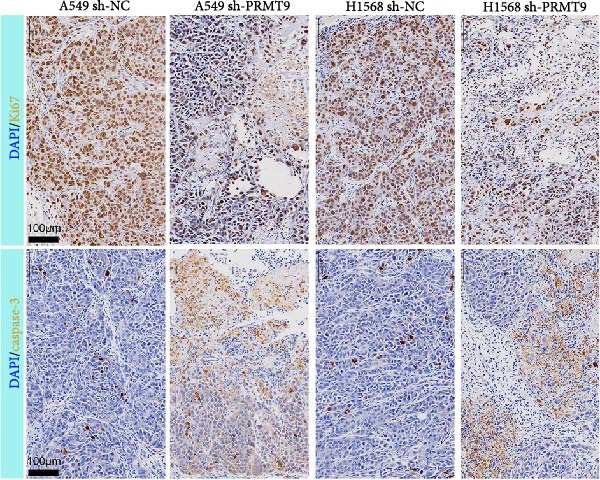
(D)

## 4. Discussion

Protein arginine methylation is a common posttranslational modification of proteins and involves the addition of methyl groups to the arginine residues of proteins. This modification is catalyzed by specific enzymes, PRMTs, which can recognize nonhistone protein targets and catalyze the addition of methyl groups onto their arginine residues [18]. Overexpression of these PRMTs is closely related to the progression of various tumors [19]. A previous study reported that inhibitors targeting PRMTs can sensitize cancer cells to various anti‐cancer drugs, including chemotherapy drugs, targeted therapy drugs, and immunotherapy drugs [20]. In our study, we found that PRMT9 was highly expressed in LUAD, and knockdown of PRMT9 significantly inhibited the proliferation, migration, and invasion of A549 and H1568 cells. Dong et al. [20] found that PRMT9 activity was elevated in the blasts and leukemia stem cells from patients with acute myeloid leukemia, and PRMT9 inhibition exerted antitumor effects by activating cyclic GMP‐AMP synthase. Shen et al. [21] reported that a PRMT9 mutation (G189R) was associated with neuronal development and that PRMT9 could regulate the arginine methylation (R508) of splicing factor 3B subunit 2 (SF3B2). In addition, PRMT9 also plays an important role in histone H4K20 monomethylation [22]. Bai et al. [23] reported that PRMT9 directly targets the signaling adaptor MAVS and catalyzes the arginine methylation of MAVS at the Arg41 and Arg43 sites. As an arginine methyltransferase, there are many target proteins of PRMT9 that may play different roles in different cells and biological processes.

PRMTs can directly interact with key signaling pathways involved in cancer progression, such as the PI3K/Akt and MAPK pathways, and thereby modulate cell survival and proliferation [24]. In hepatocellular carcinoma, PRMT9 promotes metastasis by activating PI3K/Akt/GSK‐3beta/Snail signaling [5]. Similarly, our results showed that overexpression of PRMT9 promoted the accumulation of RAS, pMek1/2, and pErk1/2 proteins, indicating that PRMT9 activates the Ras/MAPK signaling pathway. Moreover, an RAS inhibitor and MAPK inhibitor were also found to block the promoting effect of PRMT9 on LUAD cell proliferation and metastasis. Existing evidence has shown that many drugs can inhibit the malignant progression of LUAD by inactivating the Ras/MAPK signaling pathway [11, 25, 26]. A study by Lim et al. [27] suggested that hypoxia induces PRMT1 and PRMT5 expression, which subsequently increases the phosphorylation of p38 MAPK (p‐p38), phosphorylation of JNK (p‐JNK), and apoptotic molecules. However, further research is needed to show how PRMTs regulate the MAPK signaling pathway.

In the current study, we explored the reasons for the high expression of PRMT9 in LUAD tissues from two aspects: the transcriptional level and the posttranscriptional modification level. Our results showed that abnormal m6A methylation modification of PRMT9 mRNA may be one of the mechanisms leading to a high level of PRMT9. As a reader in m6A methylation modification, IGF2BP1 may be involved in recognizing m6A methylation modifications of PRMT9 mRNA and play a critical role in maintaining mRNA stability. Similar to our results, Wu et al. [28] found that IGF2BP1 was upregulated in LUAD tissues and promotes the malignant phenotypes of LUAD. Low levels of IGF2BP1 expression were found to be negatively correlated with a poor prognosis for patients with LUAD. A study by Feng et al. [29] suggested that division cycle‐associated 4 (CDCA4) activates the PI3K/AKT pathway and promotes LUAD cell proliferation by interacting with IGF2BP1. Our study revealed that knockdown of IGF2BP1 inhibits the RAS/MAPK signaling pathway in LUAD cells. Interestingly, another report confirmed that IGF2BP1 can bind to Kras RNA and that an inhibitor of IGF2BP1 induces lower Kras protein levels in cancer cells [30]. Zhao et al. [31] also found that IGF2BP1 interacts with RPS15 and directly binds the 3′‐UTR of MKK6 and MAPK14 mRNA in an m6A‐dependent manner, which promotes cancer cell metastasis and proliferation in human esophageal squamous cell carcinoma. The regulatory effect of IGF2BP1 on the MAPK signaling pathway has also been confirmed in other tumors [31, 32]. Apparently, IGF2BP1 has the potential to serve as a target for cancer treatment. In addition, other m6A‐related enzyme both involved in LUAD progression. Chen et al. [33] reported that downregulation of METTL3 promoted the proliferation, migration, and invasion of LUAD cells. Conversely, METTL14‐mediated m6A mRNA modification of G6PD promoted LUAD cell proliferation, migration, and invasion [34]. ALKBH5, as an “eraser” related to m6A modification, has been found to promote malignant progression of LUAD through regulating CDCA4 mRNA stability [35]. One limitation of our study is that we did not investigate the writers and erasers associated with the m6A modification of PRMT9 mRNA.

## 5. Conclusion

In summary, we found that PRMT9 is highly expressed in LUAD tissue and promotes cancer cell metastasis and proliferation by maintaining activation of the RAS/MAPK signaling pathway. High expression of PRMT9 was regulated by IGF2BP1—recognized m6A modification. Our research suggests PRMT9 as a target for treatment of LUAD.

**Nomenclature**
LUAD: Lung adenocarcinoma
PRMT9: Protein arginine methyltransferase 9
m6A: N6‐methyladenosine
METTL3: Methyltransferase‐like 3
PRMTs: Protein arginine methyltransferases
SF3B2: Splicing factor 3B subunit 2
p‐p38: Phosphorylation of p38 MAPK
p‐JNK: Phosphorylation of JNK
CDCA4: Cycle‐associated 4.

## Ethics Statement

All procedures performed in studies involving human participants were in accordance with the ethical standards of the institutional and/or national research committee and with the 1964 Helsinki Declaration and its later amendments or comparable ethical standards. The experiment was approved by the ethics committee of Guangzhou Twelfth People’s Hospital (Number 2024159). Procedures involving animal experiments were approved by the ethics board of Guangzhou Center for Disease Control and Prevention Experimental Animal Welfare Ethics Committee (Number 2025001).

## Consent

Informed consent was obtained from all individual participants included in the study.

## Conflicts of Interest

The authors declare no conflicts of interest.

## Author Contributions

Conceptualization, funding acquisition and writing – review and editing: Jinghua Chen. Data curation and formal analysis: Jiahao Yang and Xiangpeng Chu. Investigation and methodology: Zihao Chen. Visualization and writing – original draft: Jiahao Yang.

## Funding

This research is supported by the Guangzhou Science and Technology Planning Project (Grant 2023A03J0491).

## Supporting Information

Additional supporting information can be found online in the Supporting Information section.

## Supporting information


**Supporting Information** Table S1 Pathological TNM Staging Table for 30 Cases of Lung Adenocarcinoma.

## Data Availability

All data has been included in the manuscript and can be obtained from the corresponding author upon reasonable request.
