# Supplementary material for: m6A‐Mediated Stabilization of PRMT9 mRNA by IGF2BP1 Drives Proliferation and Metastasis in Lung Adenocarcinoma
Source: Anal Cell Pathol (Amst). 2025 Dec 10;2025:6050688. doi: 10.1155/ancp/6050688 (PMC12696393; doi:10.1155/ancp/6050688)
Supplement: Supplementary file 1 — Supporting Information Table S1 Pathological TNM Staging Table for 30 Cases of Lung Adenocarcinoma. [file ANCP-2025-6050688-s001.docx]

**Table S1** Pathological TNM Staging Table for 30 Cases of Lung Adenocarcinoma

| listings | Category | Number of people |
| --- | --- | --- |
| Gender | male | 17 |
|  | female | 13 |
| Age | >65 years old | 12 |
|  | ≤65 years old | 18 |
| Distant Metastasis (M) | M0 | 29 |
|  | M1 | 1 |
| Metastasis Quantity | Solitary | 24 |
|  | Multiple | 6 |
| TNM stage | Ⅰ | 23 |
|  | Ⅱ | 3 |
|  | Ⅲ | 4 |
|  | Ⅳ | 0 |
| Tumor size (Diameter) | >20 mm | 13 |
|  | ≤20 mm | 17 |
